# The Medical Society

**Published:** 1896-10-17

**Authors:** 


### The Medical Society.

In his Opening Presidential Address to the Medical Society on October 12th, Mr. Reginald Harrison described a mode of treatment for certain forms of albuminuria which may not improbably take a prominent place in the means at our disposal for the relief of certain cases of kidney disease. The greatly increased safety with which exploration of the kidney can now be carried out has led not unnaturally to the performance of exploratory operations in some cases in which, speaking in the grosser sense, "nothing has been found," *i.e.*, no pus, no stone, no tumour. Nevertheless, in some such cases Mr. Harrison has found that, negative as the result of the operation had seemed at the time, its ultimate result has been good—albuminuria and pain, which had before been persistent, having ceased after its performance—and he has come to the conclusion that this good result is due to the relief of tension.

In this connection he points out the disastrous results of tension in other organs, referring to intraocular tension in glaucoma and to its relief by iridectomy and allied measures, and to the striking results which in many cases follow puncture of the testicle, as recommended by the late Mr. Henry Smith, in orchitis, cases in which not only is pain relieved, but ultimate damage to the structure is prevented. However capable of gradual distension the capsule of the kidney may be, there is no doubt that it is very intolerant of any sudden increase of intra-renal tension, and experience gained in operating on that organ teaches that in certain conditions of congestion the capsule of the kidney is so tightly stretched, and its substance exposed to such pressure, as quite to explain any interference with its function. The results of operation also tend to show the importance of increased tension, for sometimes after mere incision the quantity of urine excreted is found to have doubled within twenty-four hours.

The cases in which Mr. Reginald Harrison would especially advise reni-puncture are those of acute congestion or inflammation, arising either from scarlet fever or chill, in which death seems imminent from suppression of urine, and others in which as time goes on the tendency does not seem to be towards recovery—albuminuria and casts persisting, and the functional efficiency of the kidney not being restored.

The operation itself need not be a serious one, the incision required being only moderate in extent, sufficient, that is, for digital exploration and for the making of three or four punctures, or a limited incision, according to the requirements of the case; while the conditions for which he advises it are in the one case quickly fatal, and in the other very prone to end in serious damage, not only within the kidney but throughout the blood vascular system.

